# Seasonal-Spatial Habitat Variation and Resource Status of Spear Shrimp *Mierspenaeopsis hardwickii* (Miers, 1878) in the Southern Yellow Sea and East China Sea

**DOI:** 10.3390/biology15060486

**Published:** 2026-03-19

**Authors:** Min Xu, Yong Liu, Hongmei Li, Jianzhong Ling, Huiyu Li

**Affiliations:** 1 Key Laboratory of Fisheries Remote Sensing Ministry of Agriculture and Rural Affairs, East China Sea Fisheries Research Institute, Chinese Academy of Fishery Sciences, Shanghai 200090, China; xuminwzy@aliyun.com (M.X.); liuy@ecsf.ac.cn (Y.L.); 2 Hebei Provincial Technology Innovation Center for Coastal Ecology Rahabilitation, Tangshan 063610, China; limailg@126.com

**Keywords:** beam trawl, conservation, fishery management strategy, closed area, species distribution model, hard spear shrimp, Taiwan Warm Current, Yangtze River estuary

## Abstract

*Mierspenaeopsis hardwickii* (Miers, 1878) represents an important economic resource for coastal artisanal fishers and small-scale fisheries operations. However, very little is known about the distribution patterns related to environmental factors and migration routes of *M. hardwickii*. In this study, we employed research vessels to obtain CPUE_w_ (weight in catch per unit effort) and CPUE_n_ (abundance in catch per unit effort) data in 2018–2019. Our results showed that the largest number was found at 20–40 m in spring and summer, extending in autumn (40–90 m) and shrinking in winter (40–60 m). The scattered distribution pattern of *M. hardwickii* was observed in spring with sea bottom temperature (SBT) 11–18 °C and sea bottom salinity (SBS) 32–34 and winter (SBT 9–19 °C, SBS 32–35); most individuals were observed in summer (SBT 26–28 °C, SBS 30–31) and autumn (SBT 19–22 °C, SBS 32–35). The annual mean CPUE_w_ and CPUE_n_ were 3624 g·h^–1^ and 799.4 ind·h^–1^, respectively. We hypothesize that in spring, most parent cohorts aggregate in Dasha in the southern Yellow Sea, while many cohorts gather in the coastal waters of the East China Sea, with sharply reduced abundance in the offshore deeper waters. In summer, the parent cohorts produced offspring in Lvsi in the southern Yellow Sea, the Yangtze River estuary, and coastal water areas of the East China Sea. In autumn, juveniles in the coastal waters migrated to the offshore water area. In winter, a few individuals were sparsely distributed in the offshore water areas of the southern Yellow and East China Seas, and part of the recruitment in the Taiwan Strait might migrate northward to Yushan and Wentai fishing grounds for the nursery. The present investigations provide baseline data that will enable fishers and policymakers to better manage and conserve this resource for future use.

## Simple Summary

The hard spear shrimp *Mierspenaeopsis hardwickii* (Miers, 1878) is a small-sized warm-water species with considerable potential commercial value. In Chinese coastal waters, this shrimp inhabits sandy-mud bottoms at depths of 5–90 m and has a lifespan of only one year. In recent years, overfishing has led to a marked decline in its populations in the East China Sea. The present study aims to characterize the seasonal and spatial distribution patterns of *M. hardwickii* and assess its current resource status in the southern Yellow Sea and East China Sea, with a focus on relationships with key environmental factors (i.e., depth, water temperature, and salinity). Currently, little is known about the recent resource dynamics or migration routes of this species—ecological information that is critical for the sustainable management and exploitation of small-scale coastal shrimp fisheries.

## 1. Introduction

The hard spear shrimp *Mierspenaeopsis hardwickii* (Miers, 1878) (Crustacea: Decapoda: Penaeidae: Mierspenaeopsis), an eurythermal and euryhaline warm shrimp species with small size and potentially important commercial value, inhabits sandy mud bottoms at depths of 5–90 m in China’s seas, and other areas, including Japan, Malaysia, Singapore, Pakistan, India, and Kalimantan [1]. In the southern Yellow and East China Seas of China, *M. hardwickii* extends northward from the radial sand ridge in the Subei Shoal to the Taiwan Strait [2]. *Mierspenaeopsis hardwickii* only has a one-year lifespan [3], with a maximum body length of 120 mm [4]. The diet composition of *M. hardwickii* consists of 16 taxa, including shrimps, Copepoda, Bacillariophyceae, small-sized fishes, Bivalvia, Polychaeta, Tintinnida, Foraminifera, Flagellata, Radiolaria, Gastropoda, Amphipoda, Xanthophyceae, Chlorophyceae, and Brachyura [5].

In China, the breeding period of *M. hardwickii* was suggested to be May to September via studying the ovary development, with two peaks in June–July and September, and most parent cohorts died shortly after spawning [6]. *Mierspenaeopsis hardwickii* reaches sexual maturity once a year in Zhejiang, China [2]. In India, *M. hardwickii* appears to breed throughout the fishing season, with peaks in October and April–May at Versova, Goa, and November–December at Sassoon Docks, Mumbai [7]. Moreover, in China, *M. hardwickii* might conduct short-distance east–west and north–south movements during all seasons [2].

Economically, *M. hardwickii* is an important fishing target shrimp species for the coastal and inshore fixed stake nets and beam trawls [8]. This species ranks among the top five target species in the northern waters of the East China Sea and the coastal waters shallower than 60 m in the southern East China Sea [9]. The market price of fresh shrimp generally ranges from 50 to 80 CNY per kilogram [10]. The emerging market values of shrimp can provide ample support for local, small-scale fisheries [11]. Owing to its high market price, ease of capture, and small initial expenditure for fishing gear, Tzeng (2004) suggested that the resources of marine crustaceans such as *M. hardwickii* were overfished and had clearly decreased in the East China Sea [12]. However, the recent status, migration routes, and seasonal spatial distribution patterns in relation to environmental variables of this resource in the Yellow Sea and East China Sea remain poorly understood, particularly for the period since 2008. Such ecological information is essential for the sustainable fishery management and exploitation of small-scale coastal shrimp fisheries.

On the other hand, species distribution models (SDMs) are widely employed to predict the potential distribution of crustacean species, including the quantification of spatiotemporal variations in habitat suitability across seasons and years, as well as the identification of dynamic habitat shifts under climate change scenarios [13,14]. In particular, both regression-based approaches (e.g., linear regression, generalized additive models, and multivariate discriminant analysis) and non-parametric techniques (e.g., machine learning algorithms) are applied to this end [15]. However, Araujo and New (2007) demonstrated that ensemble models outperform individual models by a considerable margin; this superiority can substantially enhance the robustness of predictions, reduce analytical biases, and thus improve the reliability of model outputs [16].

In this study, we aimed to identify the seasonal spatial distribution patterns and resource status of *M. hardwickii* in relation to environmental variables (including depth, water temperature, and salinity) in the southern Yellow Sea and East China Sea. Furthermore, we used ensemble models to predict seasonal changes in suitable habitat area and compared the predictive performance of the individual algorithms. The results of this study contribute to a better understanding of the possible migration route and the location variations in spawning and nursery grounds, and thus support coastal small-scale shrimp trawling fisheries management strategies. The findings presented in this study will aid in the sustainable conservation, restoration, and exploitation of *M. hardwickii* in the Yellow and East China Seas.

## 2. Materials and Methods

### 2.1. Sampling and Survey Procedures

**Figure 1:**
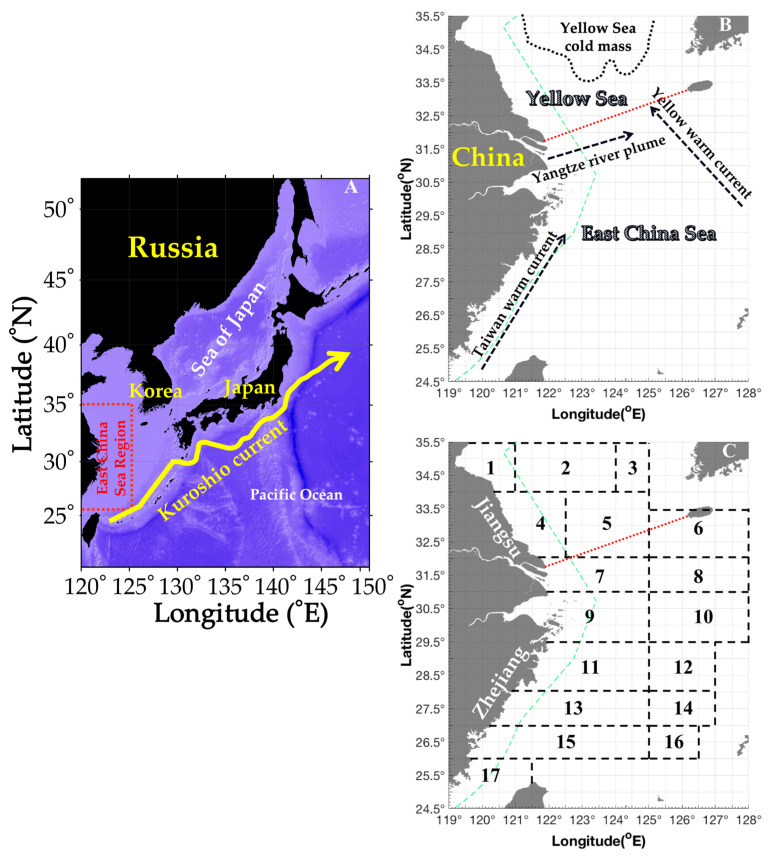
Schematic representation of the study area and survey fishing grounds. (**A**) Geographical scope of the study region (26.50° N–35.00° N, 120.00° E–127.00° E), delineated by a red dashed-line box. The bold yellow curved arrow represents the Kuroshio Current. (**B**) This area encompasses the southern Yellow Sea and East China Sea, adjacent to the coastal provinces and municipality of Fujian, Zhejiang, Jiangsu, and Shanghai. A red dashed line denotes the boundary between the Yellow Sea and the East China Sea. (**C**) Location of the 17 surveyed fishing grounds: (1) Haizhou Bay, (2) Lianqingshi, (3) Liandong, (4) Lvsi, (5) Dasha, (6) Shawai, (7) Yangtze River Estuary, (8) Jiangwai, (9) Zhoushan, (10) Zhouwai, (11) Yushan, (12) Yuwai, (13) Wentai, (14) Wenwai, (15) Mindong, (16) Minwai, and (17) Minzhong. The green dashed line indicates the motor trawling prohibition boundary.

A suite of standardized scientific bottom trawling surveys were conducted across the southern Yellow Sea and the East China Sea between 2018 and 2019, with field campaigns carried out over four distinct seasonal intervals: autumn (2–11 November 2018), winter (4–27 January 2019), spring (22 April–10 May 2019), and summer (13 August–27 September 2019) (Figure 1). The sampling gear employed was a standardized bottom trawl, characterized by a cod-end mesh size of 20 mm, a headline length of 72.24 m, a net height ranging from 10 to 15 m, and a groundline length of 82.44 m. Surveys were executed aboard the fisheries research vessels Zhongkeyu 211 and Zhongkeyu 212, covering the latitudinal range 26.50–35.00° N and longitudinal range 120.00–127.00° E. Sampling stations were positioned following a grid with a resolution of 30 arcminutes in both latitude and longitude. Each trawl was conducted at a constant speed of 3 knots, with a standard duration of 1 h per station. The final dataset included 519 valid trawling hauls, with seasonal sample sizes as follows: 127 in autumn, 111 in winter, 141 in spring, and 140 in summer.

Following trawling operations, all specimens collected were transported to the laboratory for processing to confirm species identification and document their occurrence at each sampling station. At each station, all individuals were counted, and their wet weight was measured to the nearest 0.10 g. Two catch per unit effort (CPUE) indices were calculated for the target species: biomass density (CPUE_w_, g·h^–1^) and numerical density (CPUE_n_, ind·h^–1^), both normalized to the standard 1 h trawling duration. CPUE = catch/fishing effort. Catch can be expressed as either the total weight (unit: g) or the number (unit: ind) of the catch per survey station. In this study, fishing effort refers to per hour. Average individual weight (AIW) for each station was subsequently derived as the ratio of CPUE_w_ to CPUE_n_.

A conductivity–temperature–depth (CTD) profiler (Model SBE-19, Sea-Bird Scientific, Bellevue, WA, USA) was deployed at each sampling station to collect environmental parameters. Sea surface salinity (SSS) and sea surface temperature (SST) were measured at 3 m below the sea surface, while bottom water parameters (bottom salinity, SBS; bottom temperature, SBT) were sampled at variable heights above the seafloor: 2 m for water depths < 50 m and 2–4 m for depths ≥ 50 m. Ottersen et al. (2010) highlighted that oceanographic parameters—including SST, SBT, SSS, and SBS—play a pivotal role in regulating ocean circulation patterns, vertical mixing processes, nutrient availability, and the subsequent primary productivity of marine ecosystems [17]. Collectively, these ecological processes act as key indicators and critical drivers of fluctuations in marine fishery resources [18].

### 2.2. Ensemble Model, Selection of Environmental Variables, and Evaluations

Species distribution models (SDMs) have been widely used as robust analytical tools to characterize the spatiotemporal distribution patterns and habitat suitability of marine organisms, with their implementation involving five core procedural steps: problem scoping, conceptualization, model formulation and validation, model application, and model refinement [19]. Indeed, these models are widely utilized for predicting the habitat distributions of marine fauna across China’s coastal seas and global marine regions alike Xu et al. (2025) [20].

In the present study, ten distinct algorithms were used to predict the habitat suitability of the target species based on field survey data, namely: artificial neural network (ANN), classification tree analysis (CTA), flexible discriminant analysis (FDA), generalized additive model (GAM), generalized boosting model (GBM), generalized linear model (GLM), multivariate adaptive regression splines (MARS), random forest (RF), surface range envelope (SRE), and extreme gradient boosting (XGBoost). These algorithms were integrated into a unified SDM framework to quantify and predict the relationships between species occurrence and key environmental variables (i.e., SST, SBT, SSS, SBS, and depth). Two categories of models were constructed: an annual model derived from the average values of the four seasonal survey datasets and season-specific models built using discrete seasonal data. Notably, all datasets incorporated into the modeling process were sourced exclusively from the field surveys conducted in this study.

We implemented the biomod2 R package within an ensemble species distribution model (SDM) modeling platform (4.3–4) [21]. Species occurrence data were coded as binary values (0 = absence, 1 = presence), and the full dataset was subjected to a stratified random split (80%:20%) to generate independent training and testing subsets for model calibration. All ten algorithms were constructed using a random cross-validation approach, with each algorithm independently replicated 20 times, yielding a total of 200 individual model outputs to ensure robust and stable predictive results [22]. Multicollinearity among environmental variables was eliminated by Pearson correlation analysis (|r| < 0.7) and Variance Inflation Factor (VIF) analysis (VIF < 10) prior to model construction.

Model predictive performance for each algorithm was evaluated using two widely accepted metrics: the area under the receiver operating characteristic curve (AUC) and the true skill statistic (TSS). From the 200 individual model outputs, only the top-performing models with an AUC value > 0.8 were selected, and these were further integrated into a final ensemble model using a weighted averaging method. The weighted averaging method entails computing a weighted mean of probability estimates derived from the selected individual algorithms. Specifically, these standalone algorithms are integrated into a unified ensemble model via this method, with each algorithm assigned a weight proportional to its respective performance evaluation score [23]. Detailed computational code for all modeling workflows is reported elsewhere [24]. Additional information regarding the function and practical application of the variable importance metric is available from the official biomod2 documentation (https://biomodhub.github.io/biomod2/, accessed on 15 January 2026). Habitat suitability values approaching 1 represent highly favorable conditions. A threshold of 0.7 is commonly used in species distribution modeling to delineate suitable habitat ranges, and we followed this widely accepted criterion to define suitable habitats.

## 3. Results

### 3.1. Seasonal Variation in Environmental Variables

**Figure 2:**
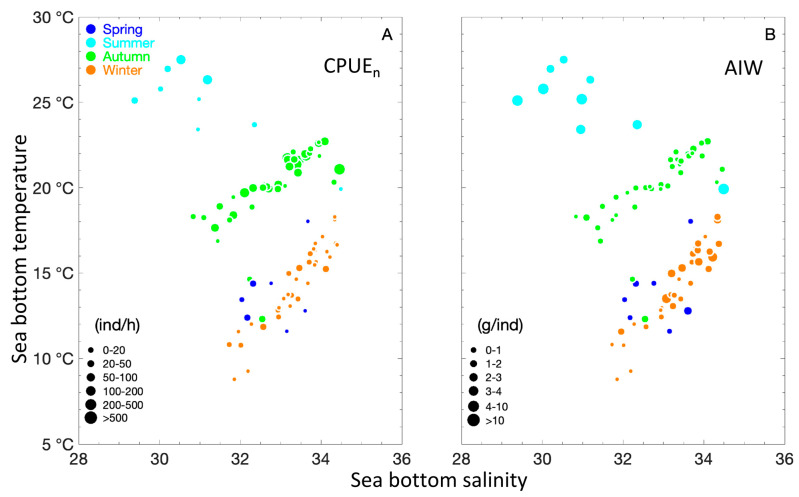
Associations between sea bottom salinity and sea bottom temperature (°C) with respect to two biological metrics of *Mierspenaeopsis hardwickii* (Miers, 1878): numerical catch per unit effort (CPUE_n_), binned into the following intervals (0–20, 20–50, 50–100, 100–200, 200–500, and >500 ind·h^−1^); and average individual weight (AIW), grouped into the categories (0–1, 1–2, 2–3, 3–4, 4–10, and >10 g·ind^−1^). Seasonal data points are represented by colored circles: blue for spring, light blue for summer, green for autumn, and brown for winter. Bivariate plot of sea bottom temperature vs. sea bottom salinity for CPUE_n_ (**A**); bivariate plot of sea bottom temperature vs. sea bottom salinity for AIW (**B**).

**Figure 3:**
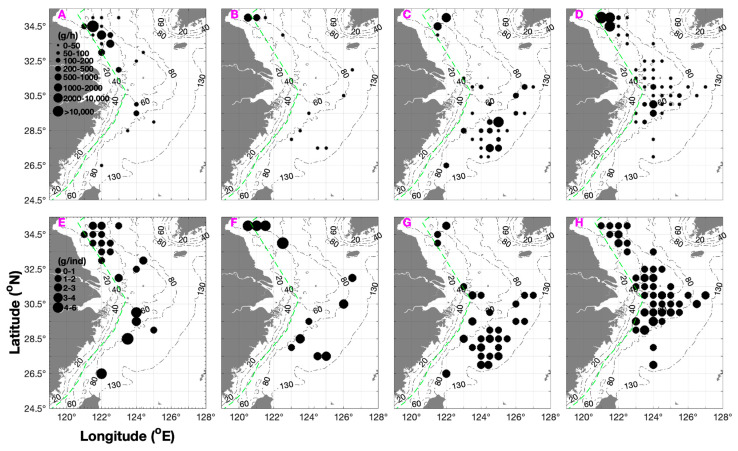
Seasonal distribution patterns of weight-based catch per unit effort (CPUE_w_; g·h^−1^) and average individual weight (AIW; g·ind^−1^) of *Mierspenaeopsis hardwickii* (Miers, 1878). Both metrics are displayed in black, with CPUE_w_ classified into the following intervals: 0–50, 50–100, 100–200, 200–500, 500–1000, 1000–2000, and >2000 g·h^−1^; AIW is grouped into 0–1, 1–2, 2–3, 3–4, 4–10, and >10 g·ind^−1^. (**A**–**D**) Seasonal CPUE_w_ distributions: (**A**) spring, (**B**) summer, (**C**) autumn, and (**D**) winter. (**E**–**H**) Seasonal AIW distributions: (**E**) spring, (**F**) summer, (**G**) autumn, and (**H**) winter. A green dashed line denotes the motor trawl ban boundary.

Most *M. hardwickii* are concentrated at depths of 20–40 m in spring, 20–30 m in summer, 40–90 m in autumn, and 40–60 m in winter (Figure 2). A scattered distribution pattern of *M. hardwickii* was observed in spring (SBT 11–18 °C, SBS 32–34) and winter (SBT 9–19 °C, SBS 32–35); most *M. hardwickii* individuals were observed in summer (SBT 26–28 °C, SBS 30–31) and autumn (SBT 19–22 °C, SBS 32–35) (Figure 3).

When AIW < 0.5 g·ind^–1^, SBT was 10–11 °C and SBS was 31–32 in winter; when AIW < 1 g·ind^–1^, SBT was 18–22 °C and 8–17 °C, and SBS was 30–35 and 32–34, respectively, in autumn and winter; when AIW > 3 g·ind^–1^, SBT was 23–28 °C and 13–19 °C, and SBS was 30–33 and 33–35 in summer and winter, respectively (Figure 3).

### 3.2. Seasonal Variation in the Resource Status

Total CPUE_w_ and CPUE_n_ were 330.5 g·h^–1^ and 162.3 ind·h^–1^ in spring, 3775.8 g·h^–1^ and 700.6 ind·h^–1^ in summer, 8809.2 g·h^–1^ and 6943.3 ind·h^–1^ in autumn, and 1580.6 g·h^–1^ and 799.4 ind·h^–1^ in winter, respectively. The seasonal ranking for total CPUE_w_ and CPUE_n_ was autumn > (summer and winter) > spring.

The annual mean CPUE_w_ and CPUE_n_ were 3624 g·h^–1^ and 799.4 ind·h^–1^, respectively (Table 1). The seasonal pattern of mean CPUE_w_ and CPUE_n_ was highest in summer and autumn, followed by spring and winter (Table 1). The seasonal order of AIW was summer > (winter and spring) > autumn (Table 1).

The longitudinal distribution of mean CPUE_w_ and CPUE_n_ was as follows: spring: 122–122.5° E > 123–125.5° E; summer: 121–122° E > 122.5–123° E; autumn: 124–126.5° E > 121.5–123.5° E; winter: 123–125.5° E > 126–126.5° E and 121.5–122.5° E (Figure 2 and Figure 3). In terms of AIW, the longitudinal distribution was spring: 122–123.5° E > 124–125.5° E; summer: 122.5–123° E > 121–122° E; autumn: 124–126.5° E > 121.5–123.5° E; and winter: 125–126.5° E > 121.5–124.5° E (Figure 3).

### 3.3. Spatial Variation in the Resource Status

In spring, CPUE_w_ and CPUE_n_ rankings for the fishing grounds were Dasha (80%) (SBT 12–14 °C, SBS 32–34, depth 20–40 m) > Yangtze River mouth (20%) (SBT 12–18 °C, SBS 32–34, depth 40–60 m), and the AIW ranking was Dasha > Yangtze River mouth. In summer, CPUE_w_ and CPUE_n_ rankings were Lvsi (80%) (SBT 23–28 °C, SBS 29–32, depth 10–30 m) > Yangtze River mouth (20%) (SBT 20–24 °C, SBS 32–35, depth 20–50 m), and the AIW ranking was Yangtze River mouth > Lvsi. In autumn, CPUE_w_ and CPUE_n_ rankings were Zhouwai (40%) (SBT 21–23 °C, SBS 34–35, depth 60–90 m) > Zhoushan (30%) (SBT 20–22 °C, SBS 33–34, depth 40–70 m) > Yangtze River mouth (~10%) (SBT 20–22 °C, SBS 32–34, depth 30–50 m) > Lvsi, Dasha, and Jiangwai (<10%) (SBT 12–23 °C, SBS 31–34, depth 10–70 m), and AIW ranking was Dasha and Zhouwai > Jiangwai > Zhoushan > Yangtze River mouth > Lvsi. In winter, CPUE_w_ and CPUE_n_ rankings were Jiangwai, Yushan, and Wentai (75%) (SBT 15–18 °C, SBS 33–35, depth 50–100 m) > Yangtze River mouth, Zhoushan, and Zhouwai (~15%) (SBT 12–17 °C, SBS 33–35, depth 40–70 m) > Lvsi and Dasha (<10%) (SBT 9–13 °C, SBS 32–33, depth 10–40 m), and AIW ranking was Yushan and Wentai > Zhouwai and Jiangwai > Zhoushan and Yangtze River mouth > Dasha > Lvsi (Figure 1 and Table 2).

### 3.4. Model Performance, Environmental Variable Importance, and Habitat Change Across the Season

**Figure 4:**
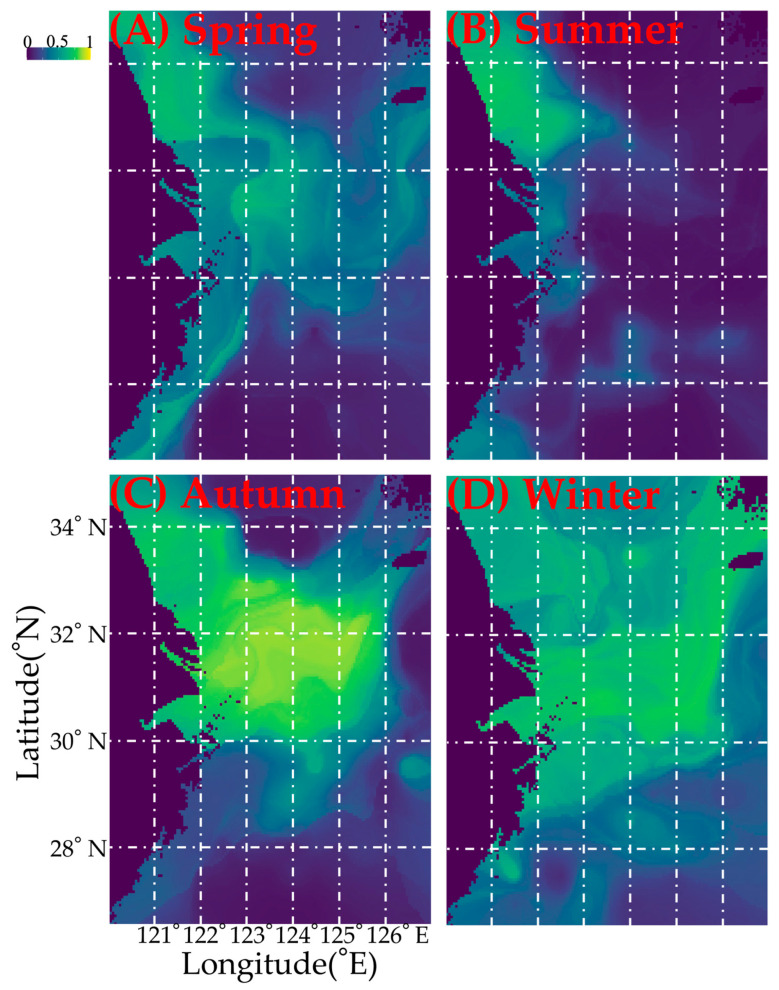
Current seasonal spatial distribution patterns of *Mierspenaeopsis hardwickii* (Miers, 1878) (panels (**A**–**D**)) in the study area, predicted from data collected during 2018–2019 and covering spring through winter.

**Figure 5:**
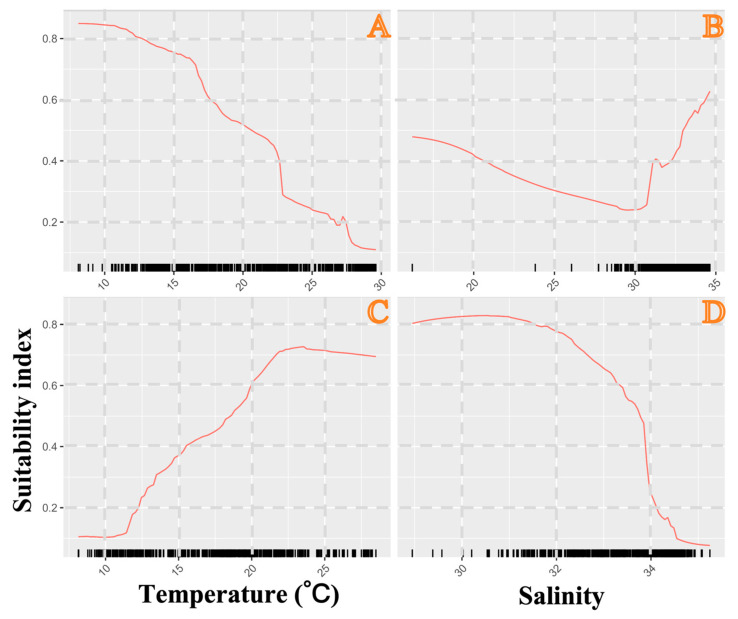
Response curves recorded by red lines of *Mierspenaeopsis hardwickii* (Miers, 1878) (panels (**A**–**D**)) to four environmental variables: (**A**) sea surface temperature (SST, 10–30 °C), (**B**) sea surface salinity (SSS, 20–35), (**C**) sea bottom temperature (SBT, 10–25 °C), and (**D**) sea bottom salinity (SBS, 30–34).

Based on the ratio of TSS to ROC values, the algorithm performance ranking was RF and GBM > CTA and XGBoost (Figure S1). In terms of ROC and TSS scores, RF and GBM were identified as the top-performing models with robust predictive performance (Figure S2). As shown in Figure S3, the most influential environmental variables varied by algorithm: sea surface temperature (SST) for ANN and GAM; sea bottom salinity (SBS) for CTA, FDA, GBM, MARS, RF, SRE, and XGBoost; and a combination of SST and SBS for GLM. Results from the ensemble model further confirmed SST and SBS as the dominant driving factors (Figure S4). Additionally, seasonal model predictions revealed clear spatiotemporal distribution patterns of *M. hardwickii*: the species was concentrated in the southern Yellow Sea and the outer waters of the Yangtze River Estuary in spring; the southern Yellow Sea (including Haizhou Bay and Lvsi) in summer; Lvsi, the Yangtze River Estuary and the Zhoushan Islands in autumn; and its distribution extended to the offshore waters of the northern East China Sea and southward to the Yushan Fishing Ground in winter (Figure 4). Our results also indicated that increases in SST and SBS may exert negative effects on the suitability of coastal habitats for *M. hardwickii*, whereas an increase in sea bottom temperature (SBT) had a positive impact (Figure 5).

## 4. Discussion

### 4.1. Migration Route in the Southern Yellow and East China Seas

In previous studies, Wu et al. (1991) suggested that most *M. hardwickii* were distributed east of 122.5° E in the Yangtze River mouth during spring and autumn and that they migrated to the coastal water area of the Yangtze River mouth from the offshore overwintering grounds between March and April [25]. In the East China Sea, with rising water temperature, parent cohorts migrated from offshore deep water to inshore shallow water for spawning in spring (March to May) [3]. The hatched juveniles fed and grew in coastal waters shallower than 30 m during summer [9]. From August to October, as the Taiwan Warm Current advanced northward, the resource center of *M*. *hardwickii* shifted northward to 31.5–33.5° N 122–126.5° E, where water temperatures ranged from 13 to 23 °C and salinity was 30–34 [3]. In autumn (November), as water temperatures decreased and juveniles grew larger, *M*. *hardwickii* gradually migrated toward offshore and southeastern waters, with higher abundances at depths of 30–60 m [3]. During winter, the resource center shifted to the offshore southeastern waters (30.5–32° N, 124–126.5° E), with water temperatures of 11–13.5 °C and salinity of 33–34. The warm current retreated southward, and the shrimp overwintered on the sandy-mud sea bottoms, with distribution extending eastward to depths of 80–100 m [3].

In the Mindongbei fishing ground of China, in spring (February), *M*. *hardwickii* migrated to the offshore southeastern sea areas along with continuous strengthening Minzhe Coastal Current and shrinking Taiwan Warm Current, in the waters of 26–27° N, 120.5–121.5° E with a water temperature of 13–21 °C and salinity of 32–34; in summer, with the prevalence of the southward monsoon, and the Minzhe Coastal Current weakens, and the Taiwan Warm Current advances northward, the aggregation center of *M*. *hardwickii* concentrated on the inshore water area with water temperature of 17–25 °C and salinity of 33.0–34.3; in autumn (November), when the northeast monsoon began to prevail in the offshore water areas, and the Minzhe Coastal Current flowing from north to south started to become strong, *M*. *hardwickii* migrated to the southward and toward the offshore water areas, mainly in 26–28° N 120–122° E with water temperature 19–25 °C and salinity 33.5–34.5 [26].

In the East China Sea, the main recruitments were found in April to July (born in the current year) and August to October (the overwintering cohorts in the previous year) [27]. Since the beginning of breeding in May, juveniles of body length 30–40 mm were present in catches from July to February of the subsequent year [3]. The dominant body length category of the female increased to 40–75 mm (83% of the stock) in September and 60–95 mm in December (85% of the stock) [3]. The minimum mean length of individuals and wet weight in September were 52.1 mm and 2 g, respectively, and the percentage of the mean length of <50 mm was ~40% [27]. By December, the mean length and body weight increased to 69.4 mm and 4.3 g, respectively [27]. Growth speed decreased in winter, and growth acceleration resumed in April of the following year [3].

**Figure 6:**
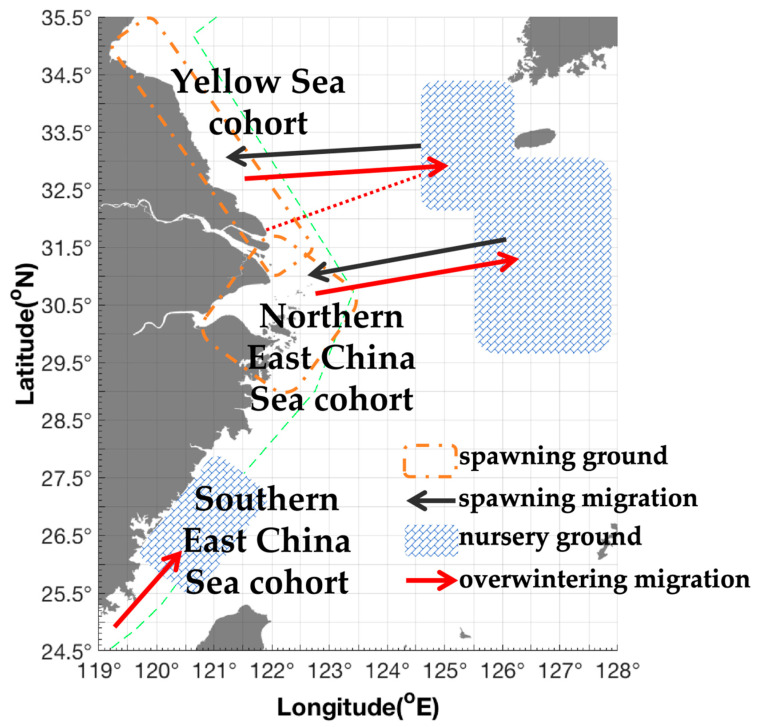
Possible migration route and spawning and nursery grounds of *Mierspenaeopsis hardwickii* (Miers, 1878).

Based on the above descriptions of the shrimp’s distribution pattern in different local water areas combined with its life history characteristics, the findings of the present study suggest that in spring most of parent cohorts aggregated in Dasha (SBT 12–14 °C, SBS 32–34) migrating from Shawai fishing grounds in the southern Yellow Sea, and part of them aggregated in Yangtze River mouth (SBT 12–18 °C, SBS 32–34) migrating from Jiangwai fishing grounds, and most cohorts aggregated in the coastal water area of the East China Sea, with sharply reduced abundance in the offshore deeper water area. In summer, the parent cohorts released the offspring in the Lvsi (SBT 23–28 °C, SBS 29–32) of the southern Yellow Sea, the Yangtze River mouth (SBT 20–24 °C, SBS 32–35) of the northern East China Sea, and coastal water areas of the East China Sea. In autumn, with water temperature decreasing, the juveniles in the coastal waters of the southern Yellow and East China Seas migrated from Lvsi and Dasha (SBT 12–23 °C, SBS 31–34) to Shawai, from the Yangtze River mouth (SBT 20–22 °C, SBS 32–34) to Jiangwai, and from the coastal area in Zhejiang to Zhouwai (SBT 21–23 °C, SBS 34–35, depth > 60 m). In winter, few individuals were sparsely distributed in the offshore water areas of the southern Yellow and East China Seas, and part of the recruitment in the Taiwan Strait might migrate northward to Yushan and Wentai fishing grounds (SBT 15–18 °C, SBS 33–35, depth > 50 m) for the spawning grounds (Table 2 and Figure 6).

### 4.2. Implications of Seasonal Spatial Variations to the Shrimp Resource

In the East China Sea, Song et al. (2009) reported the mean CPUE_w_ of 458.7 g·h^–1^ in 1998–1999, with a peak value of 1078.1 g·h^–1^ in autumn and the lowest value of 80.3 g·h^–1^ in summer [3]. In Mindongbei of Fujian, the seasonal order in 2008–2009 was as follows: August and November > February and May [26]. In this study, the mean CPUE_w_ in the southern and East China Seas was 3624 g·h^–1^, with a seasonal order of summer and autumn > spring and winter, indicating the positive impact of a summer fishing moratorium (fishing ban from 1st May to 15th September) for restoring the current biomass and preventing overexploitation on the recruitment of *M. hardwickii* in winter–spring. Our data in terms of longitudinal variations showed that the majority of cohorts were found in the coastal waters in spring–summer, and the recruitments moved to the offshore waters in autumn–winter.

Regarding the spatial distribution pattern, in the East China Sea, Song et al. (2009) found that the mean CPUE_w_ showed the order of 31–33° N (990.9 g·h^–1^) > 28–31° N (279.1 g·h^–1^) > 26–28° N (137.3 g·h^–1^) in spring, autumn, and winter; in summer, mean CPUE_w_ in 26–28° N was higher than that of 28–33°N [3]. The peak value of CPUE_w_ in 28–33° N was observed in autumn, with the lowest value in summer, and the peak value in 26–28° N was observed in summer, with the lowest value observed in spring [3]. Additionally, owing to higher water temperatures at 26–28° N, *M. hardwickii* would earlier enter coastal water for spawning [3]. In our study area, in spring and summer, *M. hardwickii* was concentrated in the southern Yellow Sea and the northern East China Sea (31–34° N), none were observed south of Yangtze River mouth fishing ground; in autumn, the mean CPUE_w_ in the area of 29.5°–31°N was twice more than that of 31–34° N, with no foundings in the south of 29.5° N; in winter, there showed the spatial order of 31–34° N (~50%) > 29.5–31° N (~25%) and 27–29.5° N (25%), indicating large spatial variations between 1998 and 1999 and 2018–2019 owing to overfishing and the implementation of a summer fishing moratorium (Table 2).

Regarding the variation in environmental factors, *M. hardwickii* was mainly distributed in 20–60 m with a water temperature of 10–24 °C and a salinity of 30–34 [3]. The majority were concentrated on the coasts of <30 m, inhabiting various substrates with depth < 70 m [3]. In Mindongbei, the occurring frequency among stations and the percentage in total CPUE_w_ were 100% and 73.4% at depths < 60 m, corresponding to 31.2% and 23.7% at depths of 60–80 m [26]. Liu et al. (1964) suggested that most *M. hardwickii* were concentrated in coastal waters with a depth of <20 m in Zhejiang in summer [28]. North of 30° N, due to the eastward expansion of the freshwater from the Yangtze River, *M. hardwickii* extended eastward to 127° E to a depth of 100 m, from a depth of 20 m, and south of 30° N, *M. hardwickii* was only distributed at depths < 70 m, with none observed east of 70 m [3]. In the present study, most were found at depths of 20–40 m in spring and summer, which extended in autumn (40–90 m), which subsequently shrank slightly in winter (40–60 m). Our water temperature and salinity data showed that most of the *M. hardwickii* stocks were impacted by the coastal freshwater input in spring and summer, and the Taiwan Warm Current might assert important roles on the migration routes of *M. hardwickii* in the southern East China Sea and Taiwan Strait in winter–spring, spring–summer, and summer–autumn.

### 4.3. Possible Fisheries Management Strategies and Plan

Fisheries management schemes are fundamental tools for the sustainable conservation and utilization of commercially important fishery resources. Understanding key aspects of a species’ life history—including seasonal spatial distribution patterns and migration behaviors—is critical to the implementation of effective management plans and the prediction of future stock potential. The penaeid prawn, *M*. *hardwickii,* is, historically, a main bycatch species for fixed stake nets and small trawlers operating in coastal waters. With the updated development of beam shrimp trawling in the southern Yellow and East China Seas since the 1980s, *M*. *hardwickii* distributed in inshore waters, has been specifically targeted for fishing [26]. In 1998, Song et al. (2009) assessed *M*. *hardwickii*‘s resource potential to be only 4000 t in Zhejiang, with a 75.8% decline compared with the early 1990s [3]. It slightly recovered to 5260.6 t in 2013, remaining 68% lower than that in the early 1990s [29]. In the present study, the mean biomass in the study area in 2018–2019 was ten times as much as that in 1998–1999, indicative of the recovery of this species under the effective protection and conservation measures.

In the East China Sea, *M*. *hardwickii* is mainly distributed in the northern East China Sea (31°–33° N) according to the shrimp trawling surveys in 1998 [3]. Another report showed that *M*. *hardwickii* is mainly distributed in the north of 31° N and the southern coastal waters of the East China Sea with depth < 60 m [8]. In the East China Sea, the fishery of *M*. *hardwickii* usually commences in late September, and the trawling fishing season mainly occurs in autumn and winter, with the fishing grounds primarily located north of 30° N [3]. In Zhejiang, China, *M*. *hardwickii* usually served as the primary target species for coastal shrimp trawling operations from January to March of the following year [30]. In Mindongbei, Fujian, China, the fishing seasons are February–May and September–November [26]. However, alternatively, it is recommended that targeted conservation measures and protection areas should be established for spawning and nursery, and some rational fishing policies should be formulated. A shrimp fishing closure area could be established in the Lvsi and Yangtze River mouth and the west of the fishing prohibition line in the East China Sea. A fishing closure period for shrimp trawling from April 1st to May 30th could be set in the aggregation ground in the study area.

Moreover, one of the main causes of decreasing *M*. *hardwickii*’s number is the intensive fishing of juveniles with a length of 30–50 mm by a large number of trawls and beam trawls [26]. The minimum legal catch size of *M*. *hardwickii* should be increased to over 70 mm [27]. When the minimum catch size is set at 70 mm, the yield per recruit reaches the maximum; when the minimum catch size is increased to 80 mm, the value per recruit attains the maximum [27]. To enable release of juveniles of length < 70 mm, it is recommended that the cod-end mesh size of single-boat beam trawls should not be <39.75 mm during the period when *M*. *hardwickii* is the primary target species [31]. It is recommended to curb the excessive increase in fishing boats, restrict legal mesh size and boat horsepower, and reduce the fishing intensities.

### 4.4. Other Management Issues

With regard to the management of fisheries stocks, it was suggested that two populations existed in the East China Sea and Taiwan Strait, and *M*. *hardwickii* in the southern East China Sea–Taiwan Strait and the northern East China Sea should be treated as two separate populations [32]. In this study, we suggested that the populations of the southern Yellow Sea, the northern East China Sea, and the southern East China Sea–Taiwan Strait should be managed in the future. The Lvsi–Dasha–Yangtze River mouth should be regarded as the main aggregation and spawning grounds in the study area for management practices.

Additionally, in recent years, climate change has been the key stressor affecting the resource status, the biological traits, and the distribution pattern of *M*. *hardwickii.* In the Zhongjieshan Archipelago of Zhejiang, *M*. *hardwickii* shifted from a common species to a dominant species between 2010 and 2018, which was suggested to be related to the increased winter catch induced by the rising sea surface temperature in recent years [2]. Surface water temperature along the eastern coast of China will exhibit a warming trend in the next 80 years, and temperature increases will be higher in summer than in winter [33]. The current trend of global anthropogenic climate change (i.e., ocean warming) and future predicted ocean CO_2_ scenarios are thought to cause widespread effects on the marine fauna, such as *M*. *hardwickii* in this study. It is necessary to carry out a long-term water temperature monitoring plan and the surveys on the current status of fishery resources.

Finally, because of the dominance of multi-species fisheries in China, it is not easy to amend the closure season for specific species or species groups. Since the 1990s, fishery management strategies in China, including vessel buyback programs, fishermen relocation programs, closed seasons and zones, the total allowable catch system, and zero and minus growth targets, have been implemented to control the effects of fishing and catch landing to prevent the decline of fishery stocks. In this study, some possible management plans, such as the fishing period of a specific species and location controls and the performance of a minimum mesh size > 70 mm, should be considered in future management practices.

## 5. Conclusions

The main conclusions of this study can be summarized as follows. Compared with two decades ago, the resource stock of *M. hardwickii* has shown a gradual recovery trend, which may be related to the impacts of climate change. The Lvsi–Yangtze River estuary and coastal waters of the East China Sea still represent the most important spawning grounds for *M. hardwickii* in spring and summer, whereas the corresponding offshore waters act as key overwintering and nursery areas in autumn and winter, with simple east–west migratory patterns. Therefore, more targeted and stricter fisheries management measures, including the designation of specific fishery closure areas and appropriate fishing moratorium periods, should be formulated and implemented to ensure the sustainable utilization of this valuable shrimp resource.

## Figures and Tables

**Table 1:** Seasonal data for the mean value and value range of catch per unit effort in weight (CPUE_w_) (unit: g·h^−1^), catch per unit effort in number (CPUE_n_) (unit: ind·h^−1^), and average individual weight (AIW) (unit: g·ind^−1^) in autumn 2018 to summer 2019.

Factor	Spring	Summer	Autumn	Winter
Mean CPUE_w_ at collection stations	47.2	419.5	231.8	46.5
Value range of CPUE_w_	2.4–122.2	51.8–883.8	1.6–2401.9	0.7–291.2
Mean CPUE_n_ at collection stations	23.2	77.8	182.7	23.5
Value range of CPUE_n_	2–59	5.5–235.6	1.3–1807.1	1–96
Mean AIW	1.9	8.1	1.3	2
Value range of AIW	1.2–3.2	3.2–14.5	0.3–2.5	0.3–6.4

**Table 2:** Mean and total values of catch per unit effort in weight (CPUE_w_) (abbreviated as B), CPUE_w_% (abbreviated as B%), catch per unit effort in number (CPUE_n_) (abbreviated as N), CPUE_n_% (abbreviated as N%), and average individual weight (abbreviated as AIW) across seasons, fishing grounds, and environmental conditions such as sea bottom temperature (abbreviated as SBT), sea bottom salinity (abbreviated as SBS), and depth in the southern Yellow and East China Seas.

Fishing Ground	Mean Value					Total Value				Environmental Variable		
	B	B%	N	N%	AIW	B	B%	N	N%	SBT	SBS	Depth
	Spring											
(5)	72.2	83.8%	33.9	79.3%	2.2	288.8	87.4%	135.7	83.6%	12.4–14.4	32.2–33.6	20–39
(7)	13.9	16.2%	8.9	20.7%	1.5	41.7	12.6%	26.6	16.4%	11.6–18	32–33.7	43–55
	Summer											
(4)	489.1	73.6%	94.2	82%	7.5	3424	90.7%	659.2	94.1%	23.4–27.5	29.4–31.2	16–28
(7)	175.9	26.4%	20.7	18%	10.2	351.8	9.3%	41.5	5.9%	19.9–23.7	32.3–34.5	18–44
	Autumn											
(4)	62.1	4.1%	67.9	5.5%	0.9	248.2	2.8%	271.6	3.9%	16.9–18.9	31.4–31.8	14–33
(5)	112.9	7.4%	86.8	7.1%	1.5	1129.1	12.8%	867.7	12.5%	12.3–20	30.8–32.6	28–65
(7)	206.6	13.6%	168.6	13.8%	1.2	2066.4	23.5%	1685.9	24.3%	19.9–21.7	32.3–33.4	35–49
(8)	119.2	7.8%	74.6	6.1%	1.4	476.9	5.4%	298.2	4.3%	20.9–22.7	33.4–34	57–69
(9)	455.4	29.9%	334.2	27.3%	1.3	3188.1	36.2%	2339.6	33.7%	20.3–22.3	33.2–34.3	41–65
(10)	566.8	37.2%	493.4	40.3%	1.5	1700.5	19.3%	1480.2	21.3%	21.1–22.7	33.9–34.5	66–84
	Winter											
(4)	4.8	1.4%	8	5%	1	19.3	1.2%	32	4%	8.8–13.1	31.9–33.2	11–33
(5)	26.8	8%	21.8	13.6%	1.3	107.1	6.8%	87.2	10.9%	10.8–12.4	31.7–32.9	29–38
(7)	43.5	13.1%	34.1	21.2%	1.7	304.7	19.3%	238.6	29.8%	11.9–13.7	32.6–33.4	35–48
(8)	91.9	27.6%	31.1	19.3%	2.6	367.6	23.3%	124.3	15.5%	14.6–18.3	33.4–34.3	57–99
(9)	35.6	10.7%	18.7	11.6%	1.9	213.5	13.5%	111.9	14%	14.4–16.7	33.7–34.4	41–66
(10)	47.6	14.3%	17.1	10.6%	2.5	238	15.1%	85.4	10.7%	15–17.1	33.2–34	58–68
(11 & 13)	82.6	24.8%	30	18.7%	3.6	330.4	20.9%	120	15%	15.2–16.7	34.1–34.4	46–78

Note: fishing grounds: (4) Lvsi, (5) Dasha, (7) Yangtze River mouth, (8) Jiangwai, (9) Zhoushan, (10) Zhouwai, (11) Yushan, and (13) Wentai. In this table, the abundance and weight data of Yushan and Wentai are combined for analysis due to their geographical proximity and the limited number of stations.

## Data Availability

The original contributions presented in this study are included in the article. Further inquiries can be directed to the corresponding author(s).

## Supplementary Materials

The following supporting information can be downloaded at https://www.mdpi.com/article/10.3390/biology15060486/s1, Figure S1: Ratio of TSS to ROC values with x- and y-directional error bars of *Mierspenaeopsis hardwickii*, generated by the artificial neural network (ANN), classification tree analysis (CTA), flexible discriminant analysis (FDA), generalized additive model (GAM), generalized boosting model (GBM), generalized linear model (GLM), multiple adaptive regression splines (MARS), random forest (RF), surface range envelope (SRE), and extreme gradient boosting (XGBoost) methods—plotted in coral red, amber orange, golden brown, vibrant grass green, teal-green, vibrant cyan, vibrant sky blue, lavender blue, vibrant magenta-pink, and vibrant hot pink, respectively; Figure S2: Calibration percentage (%) of TSS and ROC in the artificial neural network (ANN), classification tree analysis (CTA), flexible discriminant analysis (FDA), generalized additive model (GAM), generalized boosting model (GBM), generalized linear model (GLM), multiple adaptive regression splines (MARS), random forest (RF), surface range envelope (SRE), and extreme gradient boosting training (XGBOOST) methods—plotted in coral red, amber orange, golden brown, vibrant grass green, teal-green, vibrant cyan, vibrant sky blue, lavender blue, vibrant magenta-pink, and vibrant hot pink, respectively; Figure S3: Box plots of the importance of environmental variables, including sea bottom salinity (SBS), sea bottom temperature (SBT), sea surface salinity (SSS), and sea surface temperature (SST), in the artificial neural network (ANN), classification tree analysis (CTA), flexible discriminant analysis (FDA), generalized additive model (GAM), generalized boosting model (GBM), generalized linear model (GLM), multiple adaptive regression splines (MARS), random forest (RF), surface range envelope (SRE), extreme gradient boosting training (XGBOOST)—plotted in coral red, amber orange, golden brown, vibrant grass green, teal-green, vibrant cyan, vibrant sky blue, lavender blue, vibrant magenta-pink, and vibrant hot pink, respectively; Figure S4: Box plots of the importance of environmental variables, including sea bottom salinity (SBS), sea bottom temperature (SBT), sea surface salinity (SSS), and sea surface temperature (SST) for the ensemble model.
