# Supplementary material for: Seasonal-Spatial Habitat Variation and Resource Status of Spear Shrimp Mierspenaeopsis hardwickii (Miers, 1878) in the Southern Yellow Sea and East China Sea
Source: Biology (Basel). 2026 Mar 19;15(6):486. doi: 10.3390/biology15060486 (PMC13024308; doi:10.3390/biology15060486)
Supplement: Supplementary file 1 [file biology-15-00486-s001.zip › biology-4167728-supplementary.pdf]

SUPPLEMENTARY MATERIAL

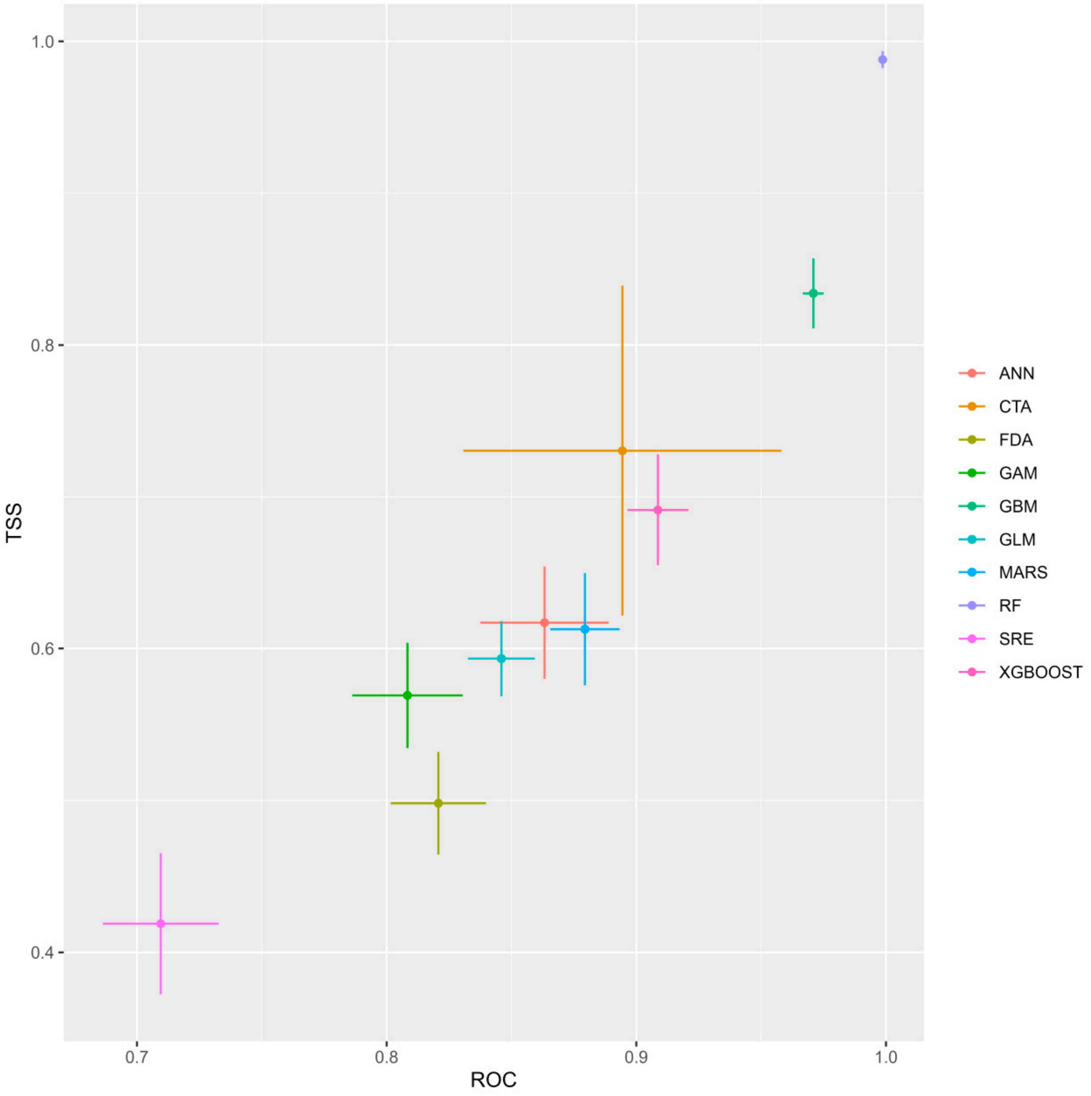

Figure S1. Ratio of TSS to ROC values with x- and y-directional error bars of *Mierspenaeopsis hardwickii*, generated by the artificial neural network (ANN), classification tree analysis (CTA), flexible discriminant analysis (FDA), generalized additive model (GAM), generalized boosting model (GBM), generalized linear model (GLM), multiple adaptive regression splines (MARS), random forest (RF), surface range envelope (SRE), and extreme gradient boosting (XGBoost) methods—plotted in coral red, amber orange, golden brown, vibrant grass green, teal-green, vibrant cyan, vibrant sky blue, lavender blue, vibrant magenta-pink, and vibrant hot pink, respectively.

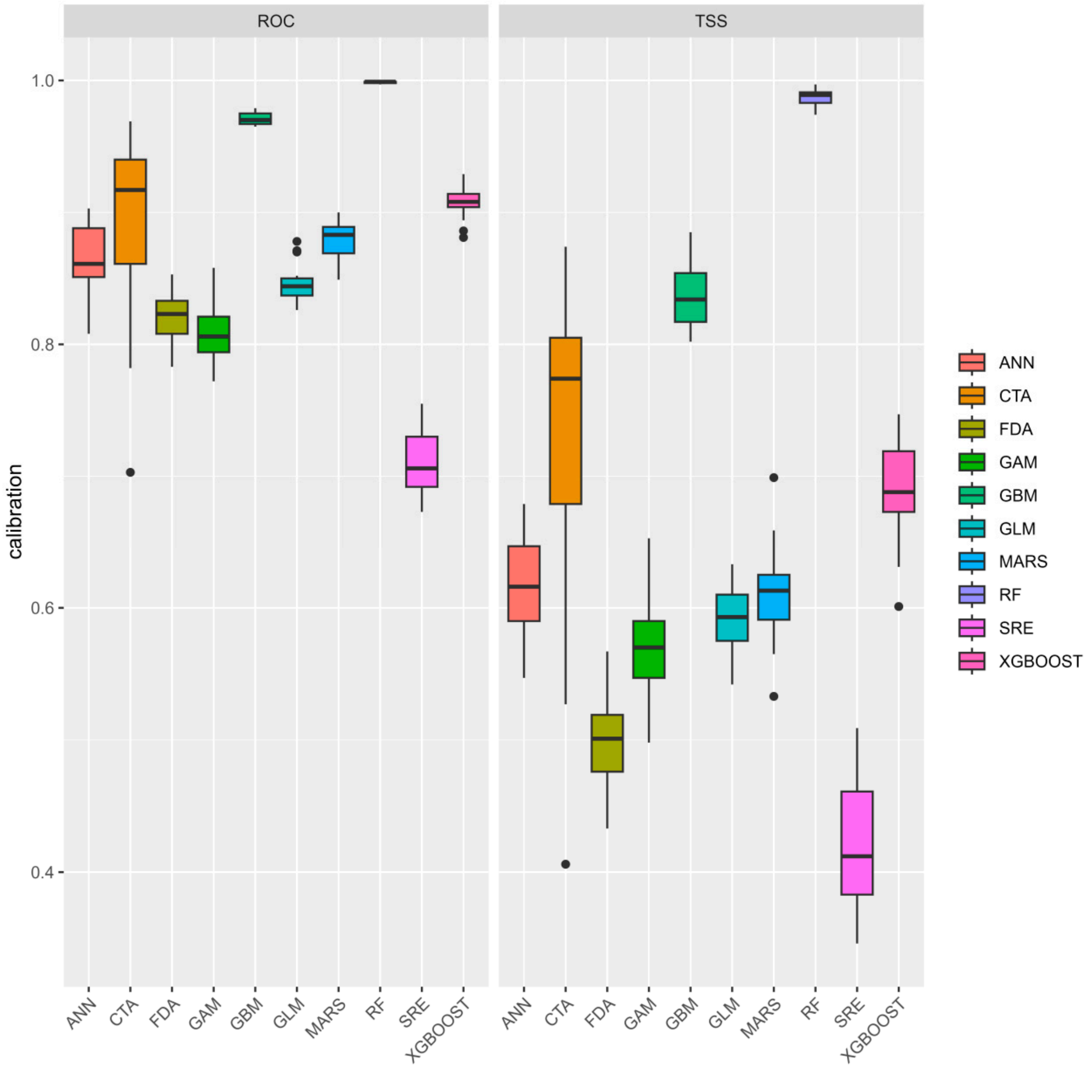

Figure S2. Calibration percentage (%) of TSS and ROC in the artificial neural network (ANN), classification tree analysis (CTA), flexible discriminant analysis (FDA), generalized additive model (GAM), generalized boosting model (GBM), generalized linear model (GLM), multiple adaptive regression splines (MARS), random forest (RF), surface range envelope (SRE), and extreme gradient boosting training (XGBOOST) methods—plotted in coral red, amber orange, golden brown, vibrant grass green, teal-green, vibrant cyan, vibrant sky blue, lavender blue, vibrant magenta-pink, and vibrant hot pink, respectively.

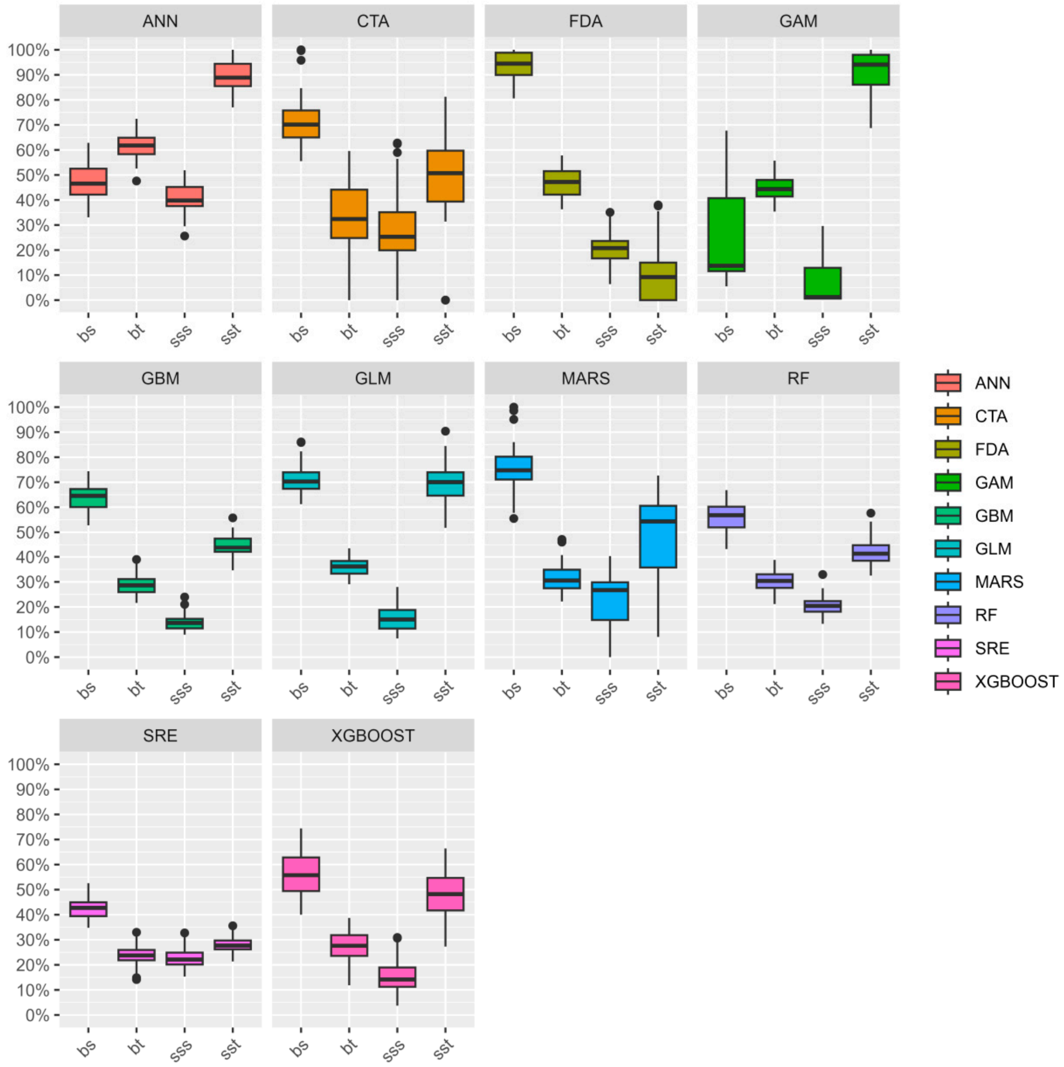

Figure S3. Box plots of the importance of environmental variables, including sea bottom salinity (SBS), sea bottom temperature (SBT), sea surface salinity (SSS), and sea surface temperature (SST), in the artificial neural network (ANN), classification tree analysis (CTA), flexible discriminant analysis (FDA), generalized additive model (GAM), generalized boosting model (GBM), generalized linear model (GLM), multiple adaptive regression splines (MARS), random forest (RF), surface range envelope (SRE), extreme gradient boosting training (XGBOOST)—plotted in coral red, amber orange, golden brown, vibrant grass green, teal-green, vibrant cyan, vibrant sky blue, lavender blue, vibrant magenta-pink, and vibrant hot pink, respectively.

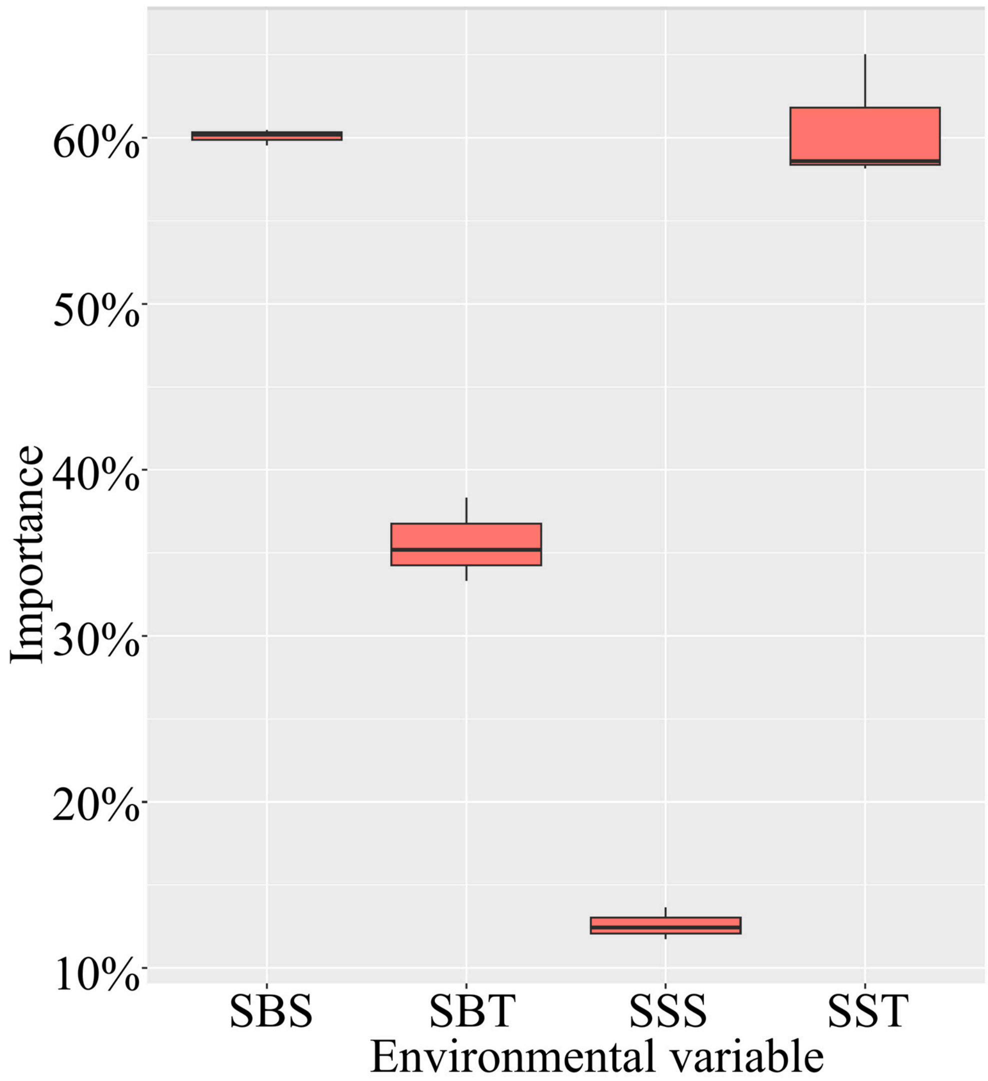

Figure S4. Box plots of the importance of environmental variables, including sea bottom salinity (bs), sea bottom temperature (bt), sea surface salinity (sss), and sea surface temperature (sst) for the ensemble model.
